# SARS-CoV-2 seroprevalence in Barcelona and Catalonia (Spain) after the 2020 lockdown

**DOI:** 10.1038/s41598-026-58310-7

**Published:** 2026-06-23

**Authors:** Candela Fernández-Naval, Aroa Silgado, Albert Blanco-Grau, Eva Dopico, Laura Calatayud, Gema Fernández-Rivas, Aroa Muñoz Quinto, Maria Teresa Sans-Mateu, Frederic F. Gómez-Bertomeu, Dúnia Perez Del Campo, Mercè Montesinos-Costa, Albert Bernet, Alba Bellés, Imma Comas, Susana Otero-Romero, Rosa María Moreno Ródenas, Nieves Larrosa, Andrés Antón, Juliana Esperalba

**Affiliations:** 1 Microbiology Department, Vall d’Hebron Hospital Universitari, Vall d’Hebron Institut de Recerca (VHIR), Barcelona, Spain; 2 Department of Microbiology, Vall d’Hebron University Hospital, Universitat Autònoma de Barcelona, PROSICS Barcelona, Barcelona, Spain; 3 Centro de Investigación Biomédica en Red de Enfermedades Infecciosas (CIBERINFEC), Instituto de Salud Carlos III, Madrid, Spain; 4 Department of Biochemistry, Hospital Universitari Vall d’Hebron, Barcelona, Spain; 5 Clinical Biochemistry, Drug Delivery and Therapy Research Group, Vall d’Hebron Research Institute (VHIR), Barcelona, Spain; 6 Microbiology Department, Hospital Universitari Bellvitge, University of Barcelona- IDIBELL, L´Hospitalet de Llobregat, Spain; 7 Microbiology Department, Clinical Laboratory North Metropolitan Area, Germans Trias i Pujol University Hospital, Badalona, Spain; 8 Departament of Genetics and Microbiology, Autonomous University of Barcelona, Badalona, Spain; 9 Clinical Analysis Laboratory ICS Camp de Tarragona-Terres de l’Ebre, Hospital Universitari Verge Cinta, Institut d’Investigació Sanitària Pere Virgili (IISPV), Tortosa, Spain; 10 Direcció Clínica Laboratoris ICS Camp de Tarragona-Terres de l’Ebre, Hospital Universitari Joan XXIII, Institut d’Investigació Sanitària Pere Virgili (IISPV), Tarragona, Spain; 11 Clinical Analysis Department, Clinical Laboratory ICS Girona, Hospital Universitari Doctor Josep Trueta, Girona, Spain; 12 Section of Microbiology, Arnau de Vilanova University Hospital Lleida, Biomedical Research Institute of Lleida, Lleida, Spain; 13 Department of Preventive Medicine and Epidemiology, Hospital Universitari Vall d’Hebron, Vall d’Hebron Institut de Recerca (VHIR), Universitat Autònoma de Barcelona, Barcelona, Spain

**Keywords:** SARS-CoV-2, Seroprevalence, COVID-19, Primary care, Leftover blood samples, Vaccine, Diseases, Health care, Medical research, Microbiology

## Abstract

The study aimed to assess seroprevalence of the severe acute respiratory syndrome coronavirus 2 (SARS-CoV-2) in Barcelona city (SRCOV2-BCN study) and the region of Catalonia, Spain (SRCOV2-CAT study) prior to the availability of coronavirus (COVID-19) vaccines. Humoral response to SARS-CoV-2 infection was assessed by detection of total antibodies (IgG, IgA and IgM) in surplus specimens from routine blood draw in the biochemistry laboratories of the participant hospitals. In Barcelona city, SARS-CoV-2 seroprevalence was 10.2% both in April and July 2020, without differences between men and women. The highest seroprevalence was found in individuals between 50 and 64 years old (13.7%). In Catalonia, SARS-CoV-2 seroprevalence in July 2020 was 9.2% and it was higher in women (10.1%) than in men (7.5%) (*P* = 0.0066), especially in the 50–64 age group (12.7% in women vs. 5.4% in men, *P* = 0.00051). The highest prevalence by age groups was 10%, found in individuals between 35 and 64 years old. Seroprevalence in the Barcelona province (11.7%) was higher than in the other provinces. In conclusion, SAR-CoV-2 seroprevalence in Catalonia during the first months of COVID pandemics was moderately high. There were differences by age, sex and geographical location that should be investigated.

**Supplementary Information:**

The online version contains supplementary material available at https://doi.org/10.1038/s41598-026-58310-7.

## Background

The severe acute respiratory syndrome coronavirus 2 (SARS-CoV-2) is the causative agent of coronavirus disease 2019 (COVID-19). First infections with SARS-CoV-2 were detected in Europe in January 2020 in travelers from Wuhan, China^1^, but later the virus spread rapidly all over the world. In Spain, state of alarm with full lockdown was officially declared on March 14, 2020^2^, was extended until the beginning of May 2020 and was followed by a period of de-escalation for gradually returning to normality on June 21, 2020. De-escalation had four phases and its timing varied by autonomous community (Spain political and administrative divisions) and even by health regions in the case of the autonomous community of Catalonia^3^. However, COVID-19 cases steadily increased during the summer of 2020 and in October 2020 a second state of alarm was declared. Vaccination began in Spain at the end of December 2020 for groups with priority and then it was implemented progressively until being widely available for Spanish population.

Catalonia is the third Spanish autonomous community by population density^4^ and as July 1^st^ 2020, Catalonia had a population of 7,741,323 individuals, from which 3,809,587 were men and 3,931,736 women^5^. Most of the Catalan population lived in the Barcelona urban area (5,227,851 individuals)^4^. Also as July 1, 2020, total population in Barcelona city was 1,634,026 individuals, from which 776,650 were men and 857,376 women^5^. All residents in Catalonia have access to public healthcare system. Regarding the evolution of COVID-19 in Catalonia, the first case was reported in Barcelona on February 25, 2020^6^. On March 11, an area of Barcelona province was confined because an outbreak was detected; on March 12 all educational facilities were closed and on March 14 full lockdown was declared. On May 15 the de-escalation began, although use of a mask was mandatory in closed spaces since May 21, and the first lockdown was finished on June 21. However, in different days of July several areas of Catalonia were confined again, including Barcelona city on day 17^7^. During the lockdowns, patients could access their health centers but only with prior appointment. The objective of the study was to describe the seroprevalence of SARS-CoV-2 infection in Barcelona city and all Catalonia after the end of the first wave of pandemics in July 2020, when vaccines were not yet available. The study comprised two substudies: SRCOV2-BCN, which included samples from both April and July 2020 to determine seroprevalence in Barcelona city; and SRCOV2-CAT, which included samples obtained in July 2020 to assess seroprevalence across Catalonia, encompassing the provinces of Barcelona, Girona, Lleida and Tarragona.

## Methods

### Study population and sample collection

Individuals visited at primary care centers of the Catalan public health system to perform a blood draw in accordance with a medical request for any biochemical marker were included consecutively, regardless of having any respiratory symptom, COVID symptoms or a polymerase chain reaction (PCR)/rapid antigen test for SARS-CoV-2, and even hospitalization for COVID-19 shortly before or after primary care visit. No exclusion criterion was defined.

The samples were surplus specimens from routine practice in the biochemistry laboratories of the participating hospitals. Analyzed samples were surplus specimens from routine practice in the biochemistry laboratories of the participating hospitals. Each participating hospital was in charge of collecting serum samples and registering demographic data (sex, age and primary care center). The minimum volume required was 1 mL per sample, although it was preferable to send 2 mL. Samples were centralized to the Vall d’Hebron University Hospital (VHUH) and should be kept refrigerated during storage and until processing.

**Fig. 1:**
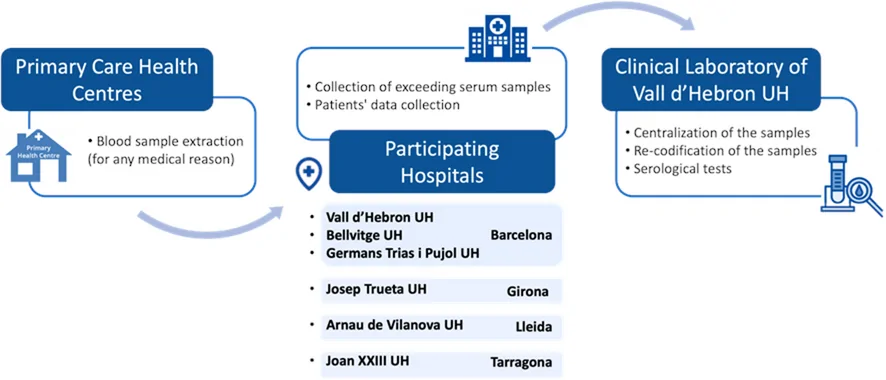
Journey of samples from collection to analysis.

In the SRCOV2-BCN substudy, samples were collected during the lockdown and at the end of the first wave of epidemics (April 2020 and July 2020, respectively). Samples were obtained from individuals who were visited at primary care centers (PPCs) located in any of the 10 municipal districts of Barcelona city and that sent to the VHUH. All samples collected over a week were analyzed together the following week for 3 days. In the SRCOV2-CAT substudy, included patients were visited at PPCs from all Catalonia in July 2020 and blood samples were sent to the corresponding referral UH (VHUH, Bellvitge UH and Germans Trias i Pujol UH in Barcelona province, Dr. Josep Trueta UH in Girona province, Arnau de Vilanova UH in Lleida province, and Joan XXIII UH in Tarragona province), according to the geographical area. Serum samples collected during a week were stored and then, the next week they were sent to the VHUH. There, in both substudies, sample containers were re-codified to allow automated serum analysis and serological tests were performed. This process is illustrated in Fig. 1.

### Sample analysis

Humoral response to SARS-CoV-2 infection was assessed by detection of total antibodies (IgG, IgA and IgM) specifically directed against the SARS-CoV-2 nucleocapsid using the Elecsys^®^ Anti-SARS-CoV-2 electrochemiluminescence immunoassay (ECLIA) (Roche Diagnostics International Ltd, Rotkreuz, Switzerland) in the instrument Cobas^®^ 801 system (Roche Diagnostics, Manheim, Germany). Results were classified according to the Cut-Off Index (COI): samples with COI < 1.0 were non-reactive (negative) and samples with COI > 1.0 were reactive (positive).

### Statistical analysis

Estimated minimum sample size was 2878 individuals, considering a population of 7,600,000 individuals, accepting an alpha risk of 0.95, with a precision of 1% and assuming a prevalence rate of 8% in the population. As some individuals might have had multiple blood draws during the periods of the substudies, datasets were analyzed searching for duplicates using medical record numbers as selection variable. Duplicates were removed to ensure that each sample represented a unique participant.

Frequencies and percentages were calculated using Microsoft Excel^®^ version 16.93. The global seroprevalence (presence of antibodies with respect to the total number of samples) and by age and sex strata in the different groups were calculated, as well as by geographical area (districts of Barcelona city and provinces of Catalonia). Comparisons between proportions were performed using the chi-square test, and differences were considered statistically significant when the *P*-value was < 0.05. For age-stratified analyses, the following age groups were used: 0–19, 20–34, 35–49, 50–64, and ≥ 65 years.

### Ethics

The Ethics Committee of the Vall d’Hebron Research Institute approved the study (PR(AG)350/2020). Consent to participate was considered not applicable because samples were surplus specimens and all patient data were anonymized according to the Spanish law on data protection^8^.

### Bias

Serum samples were obtained from individuals attending healthcare facilities for routine or clinically indicated blood tests during the study period, independent of COVID-19-related symptoms or diagnosis. This sampling strategy may introduce selection bias, as the study population may not be fully representative of the general population. In particular, individuals with chronic conditions and pregnant women may be overrepresented, as they were more likely to continue attending follow-up visits during the COVID-19 pandemic due to ongoing medical care needs and increased healthcare utilization.

## Results

In Barcelona city (SRCOV2-BCN study), a total of 906 samples were collected in April 2020; 594 (65.6%) were from women and 312 (34.4%) from men. In July 2020, a total of 1239 samples were collected, of which 770 (62.2%) samples were from women and 469 (37.9%) were from men. Out of 906 samples analyzed in April 2020, 92 (10.2%) were positive for anti-SARS-CoV-2 antibodies. There was no statistically significant difference in seroprevalence between men (10.3%) and women (10.1%). The highest prevalence was found in individuals between 50 and 64 years old (13.7%), although differences were not significant (*P* = 0.464) when compared with other age groups. In July 2020, in 1239 analyzed samples, seroprevalence was 10.2%. Difference between women (11.3%) and men (8.3%) was not statistically significant (*P* = 0.092). Seroprevalence ranged by ages from 6.5% in individuals between 20 and 34 years old to 12.1% in individuals between 35 and 49 years old, with no statistically significant differences (*P* = 0.636) (Table 1).

**Table 1:** SARS-CoV-2 seroprevalence in Barcelona in April and July 2020 by sex, age and district.

		April 2020				July 2020			
		Positive samples	Total	Sero-prevalence	*P*-value	Positive samples	Total	Sero-prevalence	*P*-value
Global		92	906	10.2%		126	1239*	10.2%	
Sex	Woman	60	594	10.1%	1.000	87	770	11.3%	0.092
	Men	32	312	10.3%		39	469	8.3%	
Age range	0–19	5	42	11.9%	0.464	6	52	11.5%	0.636
	20–34	18	205	8.8%		7	108	6.5%	
	35–49	17	189	9.0%		25	207	12.1%	
	50–64	25	182	13.7%		31	303	10.2%	
	> 65	27	288	9.4%		57	568	10.0%	
Barcelona district	Ciutat Vella	6	74	8.1%	0.234	5	82	6.1%	0.061
	Gràcia	7	60	11.7%		11	73	15.1%	
	Horta-Guinardó	7	106	6.6%		15	178	8.4%	
	L’Eixample	15	113	13.3%		28	185	15.1%	
	Les Corts	1	11	9.1%		2	39	5.1%	
	Nou Barris	15	103	14.6%		18	177	10.2%	
	Sant Andreu	13	100	13.0%		9	121	7.4%	
	Sant Martí	15	176	8.5%		24	185	13.0%	
	Sants-Montjuïc	9	144	6.3%		9	158	5.7%	
	Sarrià-Sant Gervasi	4	19	21.1%		5	41	12.2%	

*Age of 1 patient was not recorded.

In Catalonia (SRCOV2-CAT study), a total of 4280 samples were collected in July 2020, with 2752 (64.3%) samples from women and 1528 (35.7%) from men. As estimated minimum sample size was 2878 individuals, this total of samples from 4280 individuals was representative of the Catalan population. There were 392 positive samples, with a general seroprevalence of 9.2% (Table 2). When analyzed by provinces, seroprevalence was statistically significantly higher (*P* < 0.00001) in Barcelona province (11.7%) than in the other Catalan provinces (2.5% in Tarragona and 4.1% in Lleida) Table 3). Seroprevalence was higher in women (10.1%) than in men (7.5%) (*P* = 0.0066). By ages, seroprevalence ranged from 8% in individuals between 0 and 19 years old to 10% in individuals between 35 and 64 years old, with no statistically significant differences (*P* = 0.624). When analyzing data by sex and age groups, seroprevalence was higher in women across all age groups, although the differences were not statistically significant, except in the 50–64 age group (12.7% in women vs. 5.4% in men, *P* = 0.00051).

**Table 2:** SARS-CoV-2 seroprevalence in Catalonia in July 2020 by sex and age.

Age range (years)^a^	Sex	Positive samples	Total	Seroprevalence	*P*-value^b^
Global		392	4280	9.2%	
	Woman	277	2752	10.1%	0.006*
	Man	115	1528	7.5%	
0–19		**19**	**237**	**8.0%**	
	Woman	13	135	9.6%	0.418
	Man	6	102	5.9%	
20–34		**64**	**713**	**9.0%**	
	Woman	49	529	9.3%	0.761
	Man	15	184	8.2%	
35–49		**89**	**894**	**10.0%**	
	Woman	61	598	10.2%	0.053
	Man	28	296	9.5%	
50–64		**93**	**933**	**10.0%**	
	Woman	74	583	12.7%	0.00051*
	Man	19	350	5.4%	
> 65		**127**	**1496**	**8.5%**	
	Woman	80	904	8.8%	0.601
	Man	47	592	7.9%	

^a^ No age information for 7 samples; all them were negative for SARS-CoV-2.

^b^ *P*-value among the different age range groups, independently of the sex, was 0.624.

*Statistically significant difference (*P*-value < 0.05).

**Table 3:** SARS-CoV-2 seroprevalence in July 2020 by province and county.

Province	Hospital of reference	County	Positive samples	Total	Sero-prevalence	*P*-value^a^
Barcelona			356	3052	11.7%	< 0.0001
	Vall d’Hebron UH		136	1301	10.5%	
		Barcelonès	136	1301	10.5%	
	Bellvitge UH		156	1000	15.6%	
		Baix Llobregat	126	779	16.2%	
		Barcelonès	30	221	13.6%	
	Germans Trias i Pujol UH		64	725	8.8%	
		Barcelonès	36	313	11.5%	
		Maresme	13	120	10.8%	
		Vallès Occidental	4	45	8.9%	
		Vallès Oriental	11	247	4.5%	
	Dr. Josep Trueta UH (Girona)		0	26	0.0%	
		Maresme	0	26	0.0%	
Girona			**11**	**422**	**2.6%**	
	Dr. Josep Trueta UH		11	422	2.6%	
		Alt Empordà	3	102	2.9%	
		Baix Empordà	0	18	0.0%	
		Gironès	6	164	3.7%	
		La Garrotxa	0	40	0.0%	
		La Selva	2	69	2.9%	
		Pla de l’Estany	0	14	0.0%	
		Ripollès	0	13	0.0%	
		Unknown	0	2	0.0%	
Lleida			**13**	**317**	**4.1%**	
	Arnau de Vilanova UH		13	317	4.1%	
		Unknown	13	317	4.1%	
Tarragona			**12**	**489**	**2.5%**	
	Joan XXIII UH		12	489	2.5%	
		Alt Camp	1	54	1.9%	
		Baix Camp	2	126	1.6%	
		Conca de Barberà	0	13	0.0%	
		Priorat	0	8	0.0%	
		Tarragonès	9	272	3.3%	
		Unknown	0	16	0.0%	
Overall total			**392**	**4280**	**9.2%**	

UH, University Hospital.

^a^*P*-value among the different provinces (Barcelona, Girona, Lleida, Tarragona) was < 0.0001.

## Discussion

This study provides valuable insights into the seroprevalence of SARS-CoV-2 infection in Barcelona city and across Catalonia after the first wave of the pandemic, in a period when vaccines were not yet available. SARS-CoV-2 seroprevalence was assessed in a large sample of individuals in Barcelona city during the lockdown in April 2020 (SRCOV2-BCN study) as well as in all Catalonia including the city of Barcelona just after the lockdown de-escalation in July 2020 (SRCOV2-CAT study).

The results showed that seroprevalence in July 2020 was higher in the province of Barcelona (11.7%) than in the other Catalan provinces (2.5% in Tarragona and 4.1% in Lleida) (*P* < 0.00001). Seroprevalence found in Barcelona was also much higher than that reported in the Seroepidemiological Survey of SARS-CoV-2 Virus Infection in Spain (ENE-COVID), a nation-wide study^9^. In this latter, seroprevalence for the entire country was 5.0% by the rapid test and 4.6% by immunoassay, while In Catalonia, the highest seroprevalence was in Barcelona province (7.0% by rapid test and 6.8% by immunoassay). However, seroprevalence in Madrid and other six provinces of central Spain was higher than 10%^9^. This percentage was similar to that found in Barcelona city (10.2%) in April 2020 in the SRCOV2-BCN substudy of the current research as described in Table 1. Nevertheless, it should be noted that ENE-COVID and this study cannot be directly compared, because there were important differences in methodology, including timing, recruitment of participants, sample collection and laboratory tests.

Even with differences, SARS-CoV-2 seroprevalence in this study, as well as in the ENE-COVID study and other studies on seroprevalence during the first wave, was low. In Wuhan (China), from March to April 2020, seroprevalence ranged between 3.2 and 3.8%^10^. In Geneva (Switzerland), seroprevalence in April 2020 ranged from 4.8% in the first week of the study to 10.9% in the third week of the study^11^. In Los Angeles (CA, USA), in April 2020, seroprevalence was 4.6%^12^. In Northern Ireland, seroprevalence was higher during 2020–2021 but lower than 15%^13^. These results suggest that most of the non-institutionalized population was still unexposed to the virus during the first wave and after the end of the lockdown^14^. The observed differences across studies are likely to reflect, at least in part, the specific public health strategies and governmental measures implemented in each territory.

SARS-CoV-2 seroprevalence in Catalonia in 2020 was also assessed in the COVICAT study^15^, where out of 4103 individuals, 743 (18.1%) had positive serology. Of these latter, 433 (58.3%) were women. Most of participants were volunteers in a study of genomes in Catalonia (the GCAT project)^16^, while others participated in studies such as the oncological project MCC-Spain^17^ or a study in patients with chronic obstructive pulmonary disease (COPD)^18^, this latter to increase the number of older participants in COVICAT. Therefore, the selection of study population was different, as it was the collection of blood samples and the method for SARS-CoV-2 serology^15^. Furthermore, the study period was also different, because blood sample collection initiated late May 2020 and ended in November 2020^15^ and therefore blood samples were collected mainly during the de-escalation phase and the second pandemic wave in Spain, when an increase of spread of SARS-CoV-2 occurred^19^.

However, all these studies had also methodological differences in selection of study population, study time and period and serological techniques. Regarding these latter, humoral responses to SARS-CoV-2 infection can be assessed by different methods. Although to compare different methods and their results is not an objective of this study, some considerations can be made. Both in SRCOV2-BCN and SRCOV2-CAT substudies, SARS-CoV-2 infection was assessed by detection of total antibodies (IgG, IgA and IgM) specifically directed against the SARS-CoV-2 nucleocapsid using the Elecsys^®^ Anti-SARS-CoV-2 immunoassay. In the ENE-COVID study, seroprevalence was assessed with two serological tests: a point-of-care rapid blood test for qualitative differentiation between IgG and IgM against the receptor binding domain of SARS-CoV-2 spike (S) protein, and a chemiluminescent immunoassay with blood analysis in a laboratory for qualitative detection of IgG against SARS-CoV-2 nucleoprotein^9^. In the COVICAT study, multiplex quantitative suspension array technology using several SARS-CoV-2 antigens: the spike full protein (S) and the S2 fragment, the receptor-binding domain, the nucleocapsid full protein (NFL) and the nucleocapsid C-terminal region (NCt)^15^. Furthermore, it has been suggested that results of seroprevalence could depend on the prevalence of the infection in different settings, and on the pre-test probability of infection for a patient, as well as on the test accuracy (i.e., false negatives or false positives)^20^.

On the contrary, the Elecsys^®^ ECLIA was used in some studies performed during the spring or summer 2020, but mostly in patients with symptoms of COVID-1 or confirmed disease^21,22^, and aiming to validate or compare results^21–23^, not for assessing seroprevalence of SARS-CoV-2. In North Ireland in July 2020, from 1108 residual specimens from samples collected during June and July 2020, only 3.9% were positive^13^.

In April 2020, with data only from Barcelona city, SARS-CoV-2 seroprevalence was comparable between men and women. However, in July 2020, SARS-CoV-2 seroprevalence was higher in women than in men, both in the SRCOV2-BCN (11.3% and 8.3%) and SRCOV2-CAT (10.1% and 7.5%) substudies. These differences were also found in studies in Belgium and United Kingdom^24^ and in United States^25^. Seroprevalence was consistently higher in women across all age groups, although most differences were not statistically significant. Notably, a significant difference was observed in the 50–64 age group, where seroprevalence was more than twice as high in women (12.7%) compared to men (5.4%). This finding suggests potential sex- and gender-related factors influencing infection rates in this age group. We could also hypothesize that this higher prevalence in the 50–64 age group and, to a lesser degree, in the 35–49 age group, could be at least in part attributed to a higher presence of caregivers (children and older persons) in these age groups, and women are more frequently caregivers than men. Besides, epidemiological data from the Spanish Health Ministry showed also a higher prevalence of infection in women: in May 4th 2020, of 216,257 cases confirmed by PCR 121,419 (56.1%) were women and 94,835 (43.9%) were men^26^. Percentage of infected individuals between 20 and 59 years was higher in women (28.3%) than in men (18.8%)^26^. On the contrary, no difference was found in the ENECOVID study, with a positivity of 5% by rapid antigen test and 4.6% by immunoassay for both sexes^9^. Otherwise, all over the world COVID-19 was more severe in men, which had a higher risk of intensive care unit admission and death than women^27^. However, sex and gender differences in infections have not been widely assessed despite their importance^28^. For example, in a meta-analysis of 70 studies on seroprevalence of respiratory viruses other than SAR-CoV-2, possible sex differences were not assessed^29^.

Higher seroprevalences in Barcelona city and Barcelona province compared to the other Catalan provinces were probably related to the higher population density in these areas^30^, as well to increased exposure risks linked to social and occupational factors, such as a higher proportion of essential workers unable to work remotely, use of public transportation, and crowded living conditions, which collectively contribute to greater SARS-CoV-2 transmission and seroprevalence^31^.

This study has several strengths, including its sample size (4280 individuals), the wide distribution of its population across Catalonia, and its methodological approach, which offers a model for future research on seroprevalence in the general population. By using leftover patient samples from clinical routine studies for serological testing, this strategy effectively maximizes the use of available resources of clinical laboratories and may serve as a model for other infectious diseases. However, a potential improvement would be to select samples/patients by demographic data such as age and sex, which would help achieve a more balanced distribution and enhance the representativeness and reliability of the findings. However, it also had some weaknesses. First, no clinical data, prior PCR/rapid antigen test and medical record were registered and no relationship between seroprevalence and these data could be assessed. Second, study population was heterogeneous because serology screening was performed in individuals who probably had an underlying pathology or a specific condition that required a visit to a medical center during lockdown. For the same reason, participants were more likely to be old people than children. Although age information was not recorded for 7 samples, we consider that the impact on a total of 4280 samples should be minimum. Finally, no information on the place of residence (in the community or in nursing homes) was recorded, because at the time of carrying out the study, the impact of COVID-19 on mortality in Spanish nursing homes were still not completely known^32^.

The generalizability of the study findings may be limited due to the hospital-based nature of the sample, which consisted of individuals undergoing blood testing for clinical or routine reasons. As a result, the study population may not fully represent the general population of Catalonia. In particular, individuals with chronic conditions and those requiring regular medical follow-up may be overrepresented, which could influence the observed seroprevalence estimates. Nevertheless, the wide geographic coverage across Catalonia and the large sample size support the relevance of the findings for populations attending healthcare services in similar settings.

## Conclusions

SAR-CoV-2 seroprevalence in Barcelona city and in Catalonia during the first months of COVID pandemics was moderately high. There were differences by age, sex and geographical location that should be investigated; SAR-CoV-2 seroprevalence was higher in women across all age groups, with a statistically significant difference from men in the 50–64 age group, and it was also higher in Barcelona city compared to the rest of Catalonia. In addition, the methodological approach of the study could offer a model for future research on seroprevalence in the general population.

## Supplementary Information

Below is the link to the electronic supplementary material.

Supplementary Material 1

## Data Availability

The datasets generated and analyzed during the study are available from the corresponding author upon reasonable request.
