# Supplementary material for: SARS-CoV-2 seroprevalence in Barcelona and Catalonia (Spain) after the 2020 lockdown
Source: Sci Rep. 2026 Jun 23;16:28099. doi: 10.1038/s41598-026-58310-7 (PMC13554238; doi:10.1038/s41598-026-58310-7)
Supplement: Supplementary file 1 [file 41598_2026_58310_MOESM1_ESM.docx]

STROBE Statement—checklist of items that should be included in reports of observational studies

|  | Item No | | | Recommendation | ? | Comment |
| --- | --- | --- | --- | --- | --- | --- |
| **Title and abstract** | 1 | | | (*a*) Indicate the study’s design with a commonly used term in the title or the abstract | Yes | Abstract |
|  |  |  |  | (*b*) Provide in the abstract an informative and balanced summary of what was done and what was found | Yes | Abstract |
| Introduction | | | | |  |  |
| Background/rationale | 2 | | | Explain the scientific background and rationale for the investigation being reported | Yes | First paragraph |
| Objectives | 3 | | | State specific objectives, including any prespecified hypotheses | Yes | Last paragraph |
| Methods | | | | |  |  |
| Study design | 4 | | | Present key elements of study design early in the paper | Yes | Section Study population and sample collection |
| Setting | 5 | | | Describe the setting, locations, and relevant dates, including periods of recruitment, exposure, follow-up, and data collection | Yes | Section Study population and sample collection |
| Participants | 6 | | | (*a*) *Cohort study*—Give the eligibility criteria, and the sources and methods of selection of participants. Describe methods of follow-up  *Case-control study*—Give the eligibility criteria, and the sources and methods of case ascertainment and control selection. Give the rationale for the choice of cases and controls  *Cross-sectional study*—Give the eligibility criteria, and the sources and methods of selection of participants | Yes | Section Study population and sample collection |
|  |  |  |  | (*b*) *Cohort study*—For matched studies, give matching criteria and number of exposed and unexposed  *Case-control study*—For matched studies, give matching criteria and the number of controls per case |  |  |
| Variables | 7 | | | Clearly define all outcomes, exposures, predictors, potential confounders, and effect modifiers. Give diagnostic criteria, if applicable | Yes | The main outcome was seropositivity for SARS-CoV-2 antibodies, defined according to the assay cut-off provided by the manufacturer. Variables included age, sex, and geographic area of sample collection, used for descriptive analyses and stratification. |
| Data sources/ measurement | 8* | | | For each variable of interest, give sources of data and details of methods of assessment (measurement). Describe comparability of assessment methods if there is more than one group | Yes | Section Sample analysis |
| Bias | 9 | | | Describe any efforts to address potential sources of bias | Yes | Potential sources of bias were considered and are described in detail in the Methods section (Bias subsection), including possible selection bias related to the healthcare-based sampling strategy and information bias related to the serological assay performance. |
| Study size | 10 | | | Explain how the study size was arrived at | Yes | Section Statistical analysis |
| Quantitative variables | 11 | | | Explain how quantitative variables were handled in the analyses. If applicable, describe which groupings were chosen and why | Yes | Section Statistical analysis |
| Statistical methods | 12 | | | (*a*) Describe all statistical methods, including those used to control for confounding | Yes | Section Statistical analysis |
|  |  |  |  | (*b*) Describe any methods used to examine subgroups and interactions |  |  |
|  |  |  |  | (*c*) Explain how missing data were addressed |  |  |
|  |  |  |  | (*d*) *Cohort study*—If applicable, explain how loss to follow-up was addressed  *Case-control study*—If applicable, explain how matching of cases and controls was addressed  *Cross-sectional study*—If applicable, describe analytical methods taking account of sampling strategy |  |  |
|  |  |  |  | (*e*) Describe any sensitivity analyses |  |  |
| Results | | | | |  |  |
| Participants | | 13* | (a) Report numbers of individuals at each stage of study—eg numbers potentially eligible, examined for eligibility, confirmed eligible, included in the study, completing follow-up, and analysed | | Yes | Results, first and second paragraph |
|  |  |  | (b) Give reasons for non-participation at each stage | |  |  |
|  |  |  | (c) Consider use of a flow diagram | |  |  |
| Descriptive data | | 14* | (a) Give characteristics of study participants (eg demographic, clinical, social) and information on exposures and potential confounders | | Yes | Results, first and second paragraph. Tables 1-3 |
|  |  |  | (b) Indicate number of participants with missing data for each variable of interest | |  |  |
|  |  |  | (c) *Cohort study*—Summarise follow-up time (eg, average and total amount) | |  |  |
| Outcome data | | 15* | *Cohort study*—Report numbers of outcome events or summary measures over time | |  |  |
|  |  |  | *Case-control study—*Report numbers in each exposure category, or summary measures of exposure | |  |  |
|  |  |  | *Cross-sectional study—*Report numbers of outcome events or summary measures | | Yes | Results, first and second paragraph. Tables 1-3 |
| Main results | | 16 | (*a*) Give unadjusted estimates and, if applicable, confounder-adjusted estimates and their precision (eg, 95% confidence interval). Make clear which confounders were adjusted for and why they were included | | Yes | Results, first and second paragraph. Tables 1-3 |
|  |  |  | (*b*) Report category boundaries when continuous variables were categorized | | Yes | Results, first and second paragraph. Tables 1-3 |
|  |  |  | (*c*) If relevant, consider translating estimates of relative risk into absolute risk for a meaningful time period | |  |  |
| Other analyses | | 17 | Report other analyses done—eg analyses of subgroups and interactions, and sensitivity analyses | |  | Additional analyses included stratification of seroprevalence by age group, sex, and geographic area of sample collection. Found in “Statistical analysis” section. No interaction analyses or sensitivity analyses were performed. |
| Discussion | | | | |  |  |
| Key results | | 18 | Summarise key results with reference to study objectives | | Yes |  |
| Limitations | | 19 | Discuss limitations of the study, taking into account sources of potential bias or imprecision. Discuss both direction and magnitude of any potential bias | | Yes | Discussion, last paragraph |
| Interpretation | | 20 | Give a cautious overall interpretation of results considering objectives, limitations, multiplicity of analyses, results from similar studies, and other relevant evidence | |  | Results are interpreted in the Discussion section (last paragraph), where they are considered in light of study objectives, limitations (including potential selection bias), stratified analyses, and comparison with previous seroprevalence studies. |
| Generalisability | | 21 | Discuss the generalisability (external validity) of the study results | |  | Discussion section.  The external validity is limited due to the healthcare-attending nature of the study population, consisting of individuals undergoing blood testing for clinical or routine reasons, which may not fully represent the general population. However, the wide geographic coverage and large sample size support the applicability of the findings to populations attending healthcare services in similar settings. |
| Other information | | | | |  |  |
| Funding | | 22 | Give the source of funding and the role of the funders for the present study and, if applicable, for the original study on which the present article is based | | Yes | Reagents and a grant provided by Roche |

*Give information separately for cases and controls in case-control studies and, if applicable, for exposed and unexposed groups in cohort and cross-sectional studies.

**Note:** An Explanation and Elaboration article discusses each checklist item and gives methodological background and published examples of transparent reporting. The STROBE checklist is best used in conjunction with this article (freely available on the Web sites of PLoS Medicine at http://www.plosmedicine.org/, Annals of Internal Medicine at http://www.annals.org/, and Epidemiology at http://www.epidem.com/). Information on the STROBE Initiative is available at www.strobe-statement.org.
